# Assessing nurses’ professional competency: a cross-sectional study in Palestine

**DOI:** 10.1186/s12912-024-02064-y

**Published:** 2024-06-05

**Authors:** Rasha Abu Zaitoun

**Affiliations:** 1 Health Sciences Department, Faculty of Graduate Studies, Arab American University of Palestine, Ramallah Campus, Ramallah, Palestine; 2 Department of Nursing, An-Najah National University Hospital, Nablus, 44839 Palestine

**Keywords:** Nurse professional competence, Hospitals, Nursing practice, Quality of care

## Abstract

**Background:**

Evaluating nurses’ professional competence is critical for ensuring high-quality patient care. Therefore, this study aimed to evaluate the nurses’ professional competence level and to identify differences based on demographics in three West Bank hospitals.

**Methods:**

A cross-sectional design was used, and a convenient sample of 206 nurses participated in the study. The Nurse Professional Competence (NPC) Scale was used to assess the competency level. The investigator distributed the questionnaire and explained the aim of the research. Consent forms were signed before the data collection.

**Results:**

The average competency level was 79% (SD = 11.5), with 90% being professionally competent nurses. The average “nursing care” competency was 79% (SD = 12.98), and the competency level in providing value-based care was 80% (SD = 13.35). The average competency level in technical and medical care was 78% (SD = 13.45), whereas 79% (SD = 12.85) was the average competence level in “Care Pedagogics” and “Documentation and Administration “. The average competence level in the development and leadership subscale was 78% (SD = 12.22). Nurses who attended three to five workshops had a higher level of Nursing Care Competency, (H = 11.98, *p* = 0.003), and were more competent in value-based care (H = 9.29, *p* = 0.01); in pedagogical care and patient education (H = 15.16, *P* = 0.001); and in providing medical and technical care (H = 12.37, *p* = 0.002). Nurses attending more than five workshops were more competent in documentation and administration (H = 12.55, *p* = 0.002), and in development and leadership subscale ( H = 7.96, *p* = 0.20).

**Conclusion:**

The study revealed that participants lacked development and leadership skills. Engagement in workshops positively impacted the level of competencies among nurses. Notably, those attending more than five workshops exhibited greater competence in documentation, administration, development, and leadership in nursing care.

**Implications:**

This study emphasized the role of continuing education in improving nurses’ competencies and highlighted the need to conduct the study at a wider aspect to involve more hospitals with various affiliations to help structure more sensitive professional development and adopt the competencies as an integral part of staff development.

## Background

In the contemporary world, scholars prioritize the significance and function of human resources in the progress of nations; furthermore, they assert that an organization’s most critical asset is its human capital [1]. Nurses play a critical role as the primary and most valuable human resource in healthcare organizations [2]. With significant advancements in science and technology, cost control measures, and limited time for building therapeutic patient relationships, nurses are increasingly concerned about patient safety and quality of care and are committed to improving and maintaining their competencies [3].

Contemporary perspectives on professionalism underscore that enhancing the quality of healthcare is a moral and professional obligation of all medical practitioners, especially nurses. Thus, they must exhibit dedication to professional competence, transparency with patients, and improvement of care quality [4]. Professional competency is crucial in providing nursing care, and it involves adhering to professional standards [5]. The literature extensively addresses nursing competency in terms of patient safety and the quality of care provided [6].

The Novice to Expert Theory by Patricia Benner emphasizes the importance of nursing competency. Benner’s theory supports the formation of competent and trained nurses who can address the various problems of modern healthcare by offering a developmental framework, encouraging experiential learning, promoting mentorship, and improving patient safety.

skilled and knowledgeable nurses who provide high-quality care, advance patient safety, and influence good outcomes in healthcare delivery. This study is supported by Benner’s theory that emphasizes the effect of nurses’ competency on patient outcomes [7] Identifying the level of professional competency could help categorize nurses based on their level of practice and determine the proper approach to move nurses from novice to expert.

Professional competency in nurses is defined as a combination of skills, knowledge, attitudes, values, and abilities that facilitate effective performance in occupational and professional roles [8]. It involves using knowledge, technical skills, clinical reasoning, communication, emotions, and values and rethinking daily activities to provide services to individuals and society, reflecting sound judgment and habits [9].

Globally, the professional empowerment and competency of nurses are a focus of human resource management in healthcare systems, and the World Health Organization mandates that member countries report and implement plans to strengthen nurses’ competencies [10]. Nursing competency leads to improving the quality of care, increasing patient satisfaction, enhancing nursing education, and promoting nursing as a profession [11]. Patients expect competent behavior from nurses, and following the high prevalence of medical incidents, the public and media have become concerned about clinicians’ competency [12]. Thus, professionals must demonstrate their clinical competence to perform specific roles [13]. Neglecting nursing competency can cause problems for organizations, resulting in frustration, job dissatisfaction, and attrition [14].

Professional skills and competency have an impact on job attitudes, including organizational commitment and professional affiliations [15]. To achieve the goals of the healthcare system, manpower requires not only expertise, empowerment, and competency but also high levels of organizational attachment and commitment, as well as a willingness to participate in activities beyond their predetermined duties; hence, the levels of attachment and commitment of nurses to their affiliated organizations can affect the promotion of their clinical competency [1].

Nursing competency is a fundamental skill that is essential for meeting nursing obligations; hence, it is crucial to have a clear understanding of the nursing competency level to establish the basis for nursing education programs, and professional development planning and it is vital to recognize the process of nursing competency development to ensure ongoing professional growth following the acquisition of a nursing license [5]. The fundamental concept of professional competency in nursing has a direct correlation with enhancing patient care quality and safety [1].

Currently, in Palestine, there exist various levels of nurses who have graduated from a variety of nursing schools within and outside the country. Consequently, there is a diversity in their practices both at an individual and institutional level, posing a challenge to both evaluating the quality of care delivered and standardizing nursing practices nationwide. One proposed strategy to address these obstacles involves conducting an initial assessment of nurses’ competencies to establish a foundation, followed by devising a standardized professional development scheme informed by the gathered data. Unfortunately, there is a notable absence of studies that have investigated the professional competencies of nurses across different nursing specialties, leading to the absence of a comprehensive national framework for appraising nursing competencies and a lack of a standardized approach for assessing competencies.

Given that nurses are frontline healthcare providers delivering population-based health services and gatekeepers for maintaining patient safety their competency level is critical to ensure their ability to perform their daily duties effectively and efficiently to maintain high-quality care also it is an important objective method to help the nursing administrative to assess their employees level of practices and set suitable improvement plans Therefore, it is essential to measure nurses’ competency and, once measured, to establish a standard against which practice domain and performance can be evaluated. This approach provides a framework for ensuring nurses possess the necessary knowledge and skills to carry out their responsibilities effectively.

The Joint Commission Accreditation requires measuring different types of competencies based on the main patient safety goals such as infection control practices and recommends health institutions align with an organization’s strategies, business objectives, and culture for success [16]. Most commonly measured competencies verify specific nursing skills and practices and tho, but, there are limited efforts to assess the overall nursing professional competence level.

To the best of our knowledge, this study represents the first attempt to assess the level of professional competence among nurses. The choice was made to carry out this investigation within a tertiary hospital that holds accreditation from JCI. This decision was based on the premise that nurses in such settings have been immersed in a system of competency-based evaluation, potentially yielding more insightful responses compared to their counterparts in non-JCI-accredited hospitals. Furthermore, JCI-accredited hospitals typically offer ongoing professional development initiatives. The advancement of these programs requires a thorough understanding of the overall professional competence level, which is essential for structuring purposeful developmental activities.

Therefore, this study aimed to assess the level of professional competence among nurses in a tertiary hospital in the West Bank using the Nurse Professional Competence (NPC) Scale, which evaluates self-reported professional competence.

## Methods

### Study design and settings

A cross-sectional descriptive-analytic design was used to recruit the targeted participants from academic, private, and Ministry of Health hospitals. The data were collected from April to July 2023.

### Research procedure and sample

The sample was convenient to reach nurses in their place of work easily during their working time. The sample size was calculated using a Raosoft calculator (http://www.raosoft.com/samplesize.html) with a confidence level of 95%, a marginal error of 5%, and a response distribution of 50%. The estimated sample size was 286, with an attrition rate of 5%. The sample included registered nurses who provided direct patient care and had at least one year of experience in their current workplace. Head nurses, nurses who worked in the administrative field, nurses on maternal, annual, and unpaid leave, and aid nurses were excluded. Questionnaires that were completed by less than 60% of the participants were excluded from the study according to the recommendation of the original author of the tool [17]. A total of 206 nurses responded and were actively engaged in the study.

Data were gathered over a single month through the use of a self-reported, paper-based questionnaire. The questionnaire was administered in the English language because the intended participants consisted of nurses who predominantly used English for documentation and communication purposes. The questionnaires were directly distributed to the participants, allowing them to peruse the consent form, research objectives, and ethical considerations while simultaneously being encouraged to submit any inquiries they may have had. The initial page of the instrument included a description of the research aim, a consent form, and the contact information of the author.

### Research instrument

The data collection questionnaire consisted of two parts: the first part included demographic and workplace information, and the second part included the short version of the Nurse Professional Competence Scale (NPC), which was utilized to assess self-reported professional competence among nurses [18]. The Nurse Professional Competence Scale was developed by Jan Nilsson and colleagues [17] in Sweden based on Swedish national guidelines and the World Health Organization’s European Strategy for Nursing and Midwifery [19]. The original NPC scale comprises eight competency domains and a total of 88 items grouped into eight competence areas, namely, nursing care, value-based nursing care, medical and technical care, teaching/learning and support, documentation and information technology, legislation in nursing and safety planning, leadership in and development of nursing care, and education. For this study, the short version of the NPC was used [17]. The reliability and validity of the NPC Scale have been confirmed in previous studies, and the Cronbach’s alpha values of all the domains were > 0.70 [18]. .

The Nurse Professional Competence Scale has been validated and shown to have good reliability and validity in various studies conducted in the Swedish language version [20]. Responses are given on a seven-point scale ranging from a very low degree (1) to a very high degree (7), with “either high or low degree” coded as (4) [21]. The competency levels were classified into four categories based on the average score of the scale and subscales: low level (0–25), rather good level (> 25–50), good level (> 50–75), and very good level (> 75–100) [22].

For this study, Permission was obtained from the authors to use the instrument and they gave instructions to analyze the scale the instrument was piloted on 10 nurses who were excluded from the study. Some modifications were made based on the results to enhance the readability and readability of the study. The needed completion time was from 10 to 15 min.

### Analysis

The Statistical Package for the Social Sciences (SPSS) version 21 was used to analyze the data. Descriptive statistics in terms of percentage, mean and standard deviation were used to describe the demographic and work environment factors. The competency subscale scores were calculated following the formulas recommended by the author of the short version of the NPC. The nursing care competence level was calculated by summing item numbers one through 5 divided by 25 and multiplied by 100. The value-based nursing care competence level was obtained by summing the items ranging from six to ten divided by 35 and multiplied by 100. The medical and technical care competence level was estimated by summing the results of items 11 to 16 divided by 42 and multiplied by 100. The competence level in the care pedagogic was the result of summing the items ranging from 17 to 21 divided by 35 and multiplied by 100. The documentation and administrative competence level was calculated by summing the items ranging from 22 to 29 dividing by 56 and multiplying by 100; finally, the leadership and organization subscale was assessed by summing the items from 30 to 35 dividing by 42, and multiplying by 100 [17]. Moreover, the data were not normally distributed; thus, the Mann‒Whitney test and the Kruskal‒Wallis test were used to analyze the associations between demographic information and professional competency subscale scores. A *p*-value < 0.05 was considered to indicate statistical significance.

### Ethical considerations

The institutional review board of the Arab American University (AAUP) IRB NO. 2023/A/59. All nurses were given both verbal and written information about the aim and objectives of the study, and informed consent was obtained from all participants. Participants were assured that their confidentiality and anonymity would be preserved, that their participation was voluntary, and that they could withdraw at any time without any penalties.

## Results

### Demographics and work environment factors

A total of 206 nurses, with a response rate of 72%, participated in this study to assess their professional competence level. The mean age of the participants was 29.5 years, with a minimum of 21 years and a maximum of 45 years. Male nurses represented 52.4% of the participants (*n* = 108). The majority held a bachelor’s degree in nursing (*n* = 168), and 22 (10.7%) nurses held postgraduate certificates. 57% of the nurses earned a monthly income of 500–1000 JD (*n* = 57.8). 94% of the respondents received up to five courses per year. Nearly half (*n* = 94) of the participants worked as instructors for nursing students. Among those with less than six years of experience, 97 (47.1%) and 11.7% (*n* = 24) had 12 or more years of experience, respectively (Table 1).

**Table 1:** Demographics and work environment factors

Demographics	*n*	%
**Gender**		
Male	108	52.4
Female	98	47.6
**Age (years)**		
Less than 24	27	13.1
25–27	50	24.3
28–30	45	21.8
31–33	48	23.3
34–36	25	12.1
More than 37	11	5.3
**Work Place**		
Ministry of Health Hospital	8	3.9
Private Hospital	74	35.9
Academic Hospital	124	60.2
**Educational level**		
Bachelor degree	168	81.6
Associated degree	16	7.8
Postgraduate	22	10.7
**Income (Jordan Dinar)**		
Less than 500	3	1.5
500–1000	119	57.8
1000–2000	76	36.9
More than 2000	8	3.9
**Number of workshops attended**		
Less than three workshops	119	57.8
Three to five	75	36.4
More than five	12	5.8
**Work as Instructor**		
Yes	94	45.6
No	112	54.4

n: number, %: percent

### The professional competence level and subscales

Table 2 showed that the average professional competence level was 79% (SD = 11.5), with a median of 80, a minimum of 45% and a maximum of 100%. A total of 90% of the nurses were professionally competent, while 15 nurses had a competence level of less than 60%. The average “nursing care” competency was 79% (SD = 12.98), with a minimum of 34% and a maximum of 100%. The competency level of providing value-based care was 80% (SD = 13.35), with a minimum of 20% and a maximum of 100%. An average of 78% (SD = 13.45) of the participants were competent at providing technical and medical care, for a minimum of 21%. The nurses also showed an average competence level of 79% (SD = 12.85) in “Care Pedagogics”, with a minimum score of 34% and a maximum of 100%. Similarly, 79% (SD = 12.15) of the participants had an average competence level in “documentation and administration of nursing care”, for a minimum of 39%. Finally, the average competence level of the “Development, leadership and organization of Nursing Care” factor was 78% (SD = 12.22), with a minimum score of 48% and a maximum of 100% (see Table 2).

**Table 2:** Professional competence level and subscales

Professional Competence	Score (out of 100%)	SD	Minimum	Maximum
Total Competence level	79%	11.5	45%	100%
Nursing Care competence	79%	12.98	34%	100%
Value-based Care	80%	13.35	20%	100%
Technical and Medical Care	78%	13.45	21%	100%
Care Pedagogics	79%	12.85	34%	100%
Documentation and Administration of Nursing Care	79%	12.15	39%	100%
Development, leadership, and organization of Nursing Care	78%	12.22	48%	100%

SD: Standard deviation

### The difference in competency subscale scores among nurses

A significant relationship was found between the number of workshops attended by nurses and their level of competence in all competency areas. In Nursing Care, nurses who attended between three and five in-service education workshops had a higher level of Nursing Care Competency, with a mean rank of 122.39 (H = 11.98, *p* = 0.003) (see Table 3). Table 4 indicated that nurses who attended three to five workshops had a higher level of competency in applying value-based care, with a mean rank = 119.65 (H = 9.29, *p* = 0.01); in pedagogical care and patient education, with a mean rank of 123.1 (H = 15.16, *P* = 0.001) (see Table 5); and in providing medical and technical care, with a mean rank of 121.88 (H = 12.37, *p* = 0.002) (see Table 6).

**Table 3:** Differences in nursing care competence among patients according to demographic status

Nursing Care Subscale				
Demographics		Mean Rank	Median (Q1-Q3)	P*
Gender	Male	108.6	83.0 (71.0–89.0)	0.19^a^
	Female	97.3	80.0 (71.0–89.0)	
Age (years)	< 24	102.2	80.0 (71.0–86.0)	0.30^b^
	25–27	106.5	80.0 (74.0–86.0)	
	28–30	114.98	83.0 (74.0–89.0)	
	31–33	104.78	81.5 (71.0–91.0)	
	34–36	88.8	80.0 (69.0–86.0)	
	> 37	73.91	69.0 (66.0–83.0)	
Work Place	Ministry of Health Hospital	73.13	71.0 (57.75–86.0)	0.31^b^
	Private Hospital	102.69	80.0 (73.25- 86.0)	
	Academic Hospital	105.94	80.0 (71.0–89.0)	
Educational Level	Bachelor Degree	102.53	83.0 (71.75–86.0)	0.10^b^
	Associate	106.95	80.0 (71.0–88.25)	
	Post Graduate	77.86	74.0 (68.25–83.0)	
Income (Jordan Dinar)	Less than 500	58.5	71.0 (71.0–0.00)	0.30^b^
	500–1000	99.28	80.0 (71.0–86.0)	
	1000–2000	110.86	83.0 (74.0–89.0)	
	More than 2000	113.25	84.5 (67.25–93.25)	
Number of Workshops Attended	Less than three workshops	92.32	77.0 (71.0–86.0)	0.003^b^
	Three –five workshops	122.39	83.0 (77.0–94.0)	
	More than five workshops	96.38	78.5(63.75–90.5)	
Work as a Nurse Instructor	Yes	102.6	80.0(71.0–86.0)	0.85^a^
	No	104.22	80.0(71.75–86.0)	
Year of experience	Less than 6 years	100.31	80.0 (71.0–86.0)	0.19^b^
	6–11 years	111.39	83.0 (74.0–90.0)	
	12 years and more	88.44	80.0 (66.0–86.0)	

a = Mann–Whitney U test, b = Kruskal‒Wallis H test, *= significant at *p* value < 0.05

**Table 4:** Differences in value-based competences among the demographic cohorts

Values-Based				
Demographics		Mean Rank	Median (Q1-Q3)	P*
Gender	Male	101.53	81.5 (71.0–89.0)	0.62^a^
	Female	105.67	83.0 (71.0–86.0)	
Age (years)	< 24	99.39	80.0 (71.0–86.0)	0.71^b^
	25–27	111.13	83.0 (76.25–89.0)	
	28–30	104.18	83.0 (71.0–87.5)	
	31–33	104.48	84.5 (69.5–89.0)	
	34–36	100.42	83.0 (72.5–86.0)	
	> 37	78.86	74.0 (57.0–89.0)	
Work Place	Ministry of Health Hospital	68.44	70.0 (59.25–86.0)	0.18^b^
	Private Hospital	100.84	83.0 (71.0–86.0)	
	Academic Hospital	107.35	83.0 (71.0–89.0)	
Educational Level	Bachelor Degree	109.22	83.0 (71.0–86.0)	0.12^b^
	Associate	106.24	84.5 (71.25–88.25)	
	Post Graduate	78.39	75.5 (65.25–83.0)	
Income (Jordan Dinar)	Less than 500	78.67	71.0 971.0–0.00)	0.15^b^
	500–1000	96.84	80.0 (71.0–6.0)	
	1000–2000	112.03	83.0 (74.75–89.0)	
	More than 2000	130.94	84.5 (67.25–93.25)	
Number of Workshops Attended	Less than three workshops	89.81	80.0 (71.0–86.0)	0.001^b^
	Three –five workshops	123.01	86.0 (77.0–94.0)	
	More than five workshops	117.29	86.0 (71.0–93.25)	
Work as a Nurse Instructor	Yes	98.03	80.0 (71.0–86.0)	0.26^a^
	No	108.09	83.0 (71.0–89.0)	
Year of experience	Less than 6 years	103.78	83.0 (71.0–86.0)	0.97^b^
	6–11 years	1.3.94	83.0 (71.0–86.0)	
	12 years and more	100.81	84.5 (71.75–86.0)	

a = Mann–Whitney U test, b = Kruskal‒Wallis H test, *= significant at *p* value < 0.05

**Table 5:** Differences in pedagogical competency subscale among patients according to demographic status

Pedagogic Competency Subscale				
Demographics		Mean Rank	Median (Q1-Q3)	P*
Gender	Male	104.29	83.0 (69.5–86.0)	0.84^a^
	Female	102.63	80.0 (71.0–86.0)	
Age (years)	< 24	89.41	77.0 (69.0–86.0)	0.40^b^
	25–27	99.88	78.5 (70.5–86.0)	
	28–30	110.23	83.0 (71.0- 87.5)	
	31–33	113.17	86.0 (71.0–88.25)	
	34–36	105	83.0 (71.0–86.0)	
	> 37	80.95	77.0 (63.0–86.0)	
Work Place	Ministry of Health Hospital	91.13	84.5 (61.5–86.0)	0.83^b^
	Private Hospital	103.67	83.0 (71.0–86.0)	
	Academic Hospital	104.2	80.0 (71.0–86.0)	
Educational Level	Bachelor Degree	121.94	83.0 (71.0–86.0)	0.15^b^
	Associate	104.2	86.0 (74.0–92.0)	
	Post Graduate	84.75	72.5 (63.0–86.0)	
Income (Jordan Dinar)	Less than 500	71.33	71.0 (57.0–0.0)	0.33^b^
	500–1000	98.48	80.0 (71.0–86.0)	
	1000–2000	111.28	83.0 (71.0–89.0)	
	More than 2000	116.25	86.0 (71.75–86.0)	
Number of Workshops Attended	Less than three workshops	93.11	80.0 (69.0–86.0)	0.01^b^
	Three –five workshops	119.65	86.0 (71.0–94.0)	
	More than five workshops	105.58	80.0 (67.25–89.0)	
Work as a Nurse Instructor	Yes	97.54	80.0 (70.5–86.0)	0.19^a^
	No	108.5	83.0 (71.0–86.0)	
Year of experience	Less than 6 years	98.1	80.0 (70.0–86.0)	0.13^b^
	6–11 years	113.12	86.0 (71.0–89.0)	
	12 years and more	91.25	81.5 (69.0–86.0)	

a = Mann–Whitney U test, b = Kruskal‒Wallis H test, *= significant at *p* value < 0.05

**Table 6:** Differences in medical and technological competencies among patients according to demographic status

Medical and Technological Care				
Demographics		Mean Rank	Median (Q1-Q3)	P*
Gender	Male	105.81	81.0 (69.0–86.0)	0.56^a^
	Female	100.9	80.0 (69.0–86.0)	
Age (years)	< 24	85.63	76.0 (64.0–86.0)	0.22^b^
	25–27	105.15	81.0 (71.0–86.0)	
	28–30	113.1	86.0 (69.0–87.0)	
	31–33	113.14	86.0 (71.75–86.0)	
	34–36	92.68	79.0 (65.5–86.0)	
	> 37	83.14	69.0 (60.0–88.0)	
Work Place	Ministry of Health Hospital	90.06	80.0 (60.25–86.0)	0.81^b^
	Private Hospital	103.66	81.0 (70.5–86.0)	
	Academic Hospital	104.27	81.0 (69.0–86.0)	
Educational Level	Bachelor Degree	111.5	81.0 (71.0–86.0)	0.25^b^
	Associate	105.26	82.0 (69.0–89.0)	
	Post Graduate	84.25	73.5 (67.75–86.5)	
Income (Jordan Dinar)	Less than 500	68.83	69.0 (57.0–0.00)	0.32^b^
	500–1000	99.23	81.0 (69.0–86.0)	
	1000–2000	109.2	81.0 (71.75–88.0)	
	More than 2000	125.88	86.0 (71.5–93.0)	
Number of Workshops Attended	Less than three workshops	91.31	79.0 (69.0–86.0)	0.002^b^
	Three –five workshops	121.88	86.0 (76.0–90.0)	
	More than five workshops	109.54	86.0 (68.0–86.0)	
Work as a Nurse Instructor	Yes	100.8	81.0 (69.0–86.0)	0.55^a^
	No	105.77	81.0 (69.5–86.0)	
Year of experience	Less than 6 years	97.26	79.0 (69.0–86.0)	0.17^b^
	6–11 years	112.74	86.0 (71.0–88.0)	
	12 years and more	96	80. (67.5–86.0)	

a = Mann–Whitney U test, b = Kruskal‒Wallis H test, *= significant at *p* value < 0.05

Table 7 revealed that nurses who attended more than five workshops were more competent in documenting and administering nursing care, with a mean rank of 130.0 (H = 12.55, *p* = 0.002), and in developing and leading nursing care (mean rank = 121.7, H = 7.96, *p* = 0.20) (see Table 8). Similarly, Table nine shows that attending three to five workshops was associated with a higher total professional competence level, with a mean rank of 121.05 (H = 12.11, *p* = 0.002). However, there were no significant differences in the total professional competency level or other professional competency subscale scores among other demographic and work environment factors (see Table 9). The reliability of the short version of the questionnaire in this study was excellent, with a Cronbach’s alpha of 97%.

**Table 7:** Differences in documentation competency subscale among patients according to demographic status

Documentation and Administration				
Demographics		Mean Rank	Median (Q1-Q3)	P*
Gender	Male	106.2	82.0 (71.0-87.5)	0.49^a^
	Female	100.53	81.0 (71.0–86.0)	
Age (years)	< 24	88.41	75.0 (63.0–86.0)	0.65^b^
	25–27	102.28	80.0 (71.0–86.0)	
	28–30	109.89	82.0 (71.0–89.5)	
	31–33	110.14	86.0 (71.0–88.0)	
	34–36	103.08	82.0 (72.0–86.0)	
	> 37	91.95	79.0 (64.0–86.0)	
Work Place	Ministry of Health Hospital	80.19	78.5 (61.25–86.0)	0.38^b^
	Private Hospital	108.89	86.0 (71.0–88.0)	
	Academic Hospital	101.79	80.0 (71.0–86.0)	
Educational Level	Bachelor Degree	114.03	82.0 (71.0–86.0)	0.41^b^
	Associate	104.34	86.0 (72.0–87.5)	
	Post Graduate	89.43	78.0 (63.75–86.5)	
Income (Jordan Dinar)	Less than 500	68.67	71.0 (61.0–0.0)	0.35^b^
	500–1000	99.1	80.0 (71.0–86.0)	
	1000–2000	109.96	85.0 (71.5–88.0)	
	More than 2000	120.63	86.0 (73.25–91.75)	
Number of Workshops Attended	Less than three workshops	91.2	79.0 (70.0–86.0)	0.002^b^
	Three –five workshops	118.77	86.0 (73.0–89.0)	
	More than five workshops	130	87.0 (74.0–92.5)	
Work as a Nurse Instructor	Yes	103.6	82.0 (71.0–86.0)	0.98^a^
	No	103.42	81.0 (71.0- 87.5)	
Year of experience	Less than 6 years	99.42	80.0 (71.0–86.0)	0.57^b^
	6–11 years	108.63	86.0 (71.0–88.0)	
	12 years and more	101.81	84.0 (73.5–86.0)	

a = Mann–Whitney U test, b = Kruskal‒Wallis H test, *= significant at *p* value < 0.05

**Table 8:** Differences in development competency subscale among patients according to demographics

Development and Leadership				
Demographics		Mean Rank	Median (Q1-Q3)	P*
Gender	Male	106.97	79.0 (71.0–86.0)	0.38^a^
	Female	99.68	79.0 (71.0–86.0)	
Age (years)	< 24	78.48	74.0 (62.0–79.0)	0.25^b^
	25–27	106.64	80.0 (71.0–86.0)	
	28–30	110.79	79.0 (71.0–90.5)	
	31–33	106.61	80.0 (71.75–86.0)	
	34–36	110.66	83.0 (74.0–86.0)	
	> 37	90.95	79.0 (55.0–86.0)	
Work Place	Ministry of Health Hospital	97.88	81.0 (67.0–86.0)	0.90^b^
	Private Hospital	101.7	79.0 (67.75–86.0)	
	Academic Hospital	104.94	79.0 (71.0–86.0)	
Educational Level	Bachelor Degree	102.5	79.0 (71.0–86.0)	0.87^b^
	Associate	104.41	77.5 (71.0–86.0)	
	Post Graduate	97.27	79.0 (64.0–86.5)	
Income (Jordan Dinar)	Less than 500	77.33	74.0 (57.0–0.0)	0.19^b^
	500–1000	97.03	79.0 (67.0–86.0)	
	1000–2000	112.53	83.0 (74.0–86.0)	
	More than 2000	123.81	82.5 (73.0–90.0)	
Number of Workshops Attended	Less than three workshops	93.6	79.0 (67.0–86.0)	0.02^b^
	Three –five workshops	116.3	83.0 (74.0–88.0)	
	More than five workshops	121.71	84.0 (70.75–93.75)	
Work as a Nurse Instructor	Yes	105.19	79.0 (71.0–86.0)	0.71^a^
	No	102.08	79.0 (71.0–86.0)	
Year of experience	Less than 6 years	101.57	79.0 (71.0–86.0)	0.89^b^
	6–11 years	105.78	79.0 (71.0–86.0)	
	12 years and more	103.21	79.0 (71.75–86.0)	

a = Mann–Whitney U test, b = Kruskal‒Wallis H test, *= significant at *p* value < 0.05

**Table 9:** Differences in total competency scale among patients according to demographics

Competence Score				
Demographics		Mean Rank	Median (Q1-Q3)	P*
Gender	Male	106.01	81.0 (71.0–88.0)	0.53^a^
	Female	100.73	79.0 (71.0–86.0)	
Age (years)	< 24	90.07	78.0 (67.0–86.0)	0.51^b^
	25–27	105.19	79.0 (73.0–86.0)	
	28–30	112.36	82.0(72.5–88.5)	
	31–33	108.45	84.0 (70.25–87.75)	
	34–36	98.26	79.0 (69.0–86.0)	
	> 37	82.86	75.0 (60. − 86.0)	
Work Place	Ministry of Health Hospital	82.19	77.5 (62.0–86.0)	0.58^b^
	Private Hospital	103.45	82.0 (72.0–86.0)	
	Academic Hospital	104.91	79.5 (71.0–87.0)	
Educational Level	Bachelor Degree	109.88	81.0 (71.0–87.0)	0.21^b^
	Associate	105.65	82.5 (72.0–86.0)	
	Post Graduate	82.48	75.0 (66.0–83.0)	
Income (Jordan Dinar)	Less than 500	67.5	71.0 (62.0–0.0)	0.16^b^
	500–1000	97.46	78.0 (71.0–86.0)	
	1000–2000	111.74	82.5 (72.25–88.0)	
	More than 2000	128.56	86.5 (73.75–88.0)	
Number of Workshops Attended	Less than three workshops	91.19	79.0 (70.0–85.0)	0.002^b^
	Three –five workshops	121.05	84.0 (75.0–91.0)	
	More than five workshops	115.92	86.5 (67.5–88.0)	
Work as a Nurse Instructor	Yes	101.07	80.0 (71.0–86.0)	0.59^a^
	No	105.54	80.5 (71.0–87.0)	
Year of experience	Less than 6 years	99.14	79.0 (71.0–86.0)	0.39^b^
	6–11 years	110.22	84.0 (71.0–88.0)	
	12 years and more	97.31	79.0 (70.75–86.0)	

a = Mann–Whitney U test, b = Kruskal‒Wallis H test, *= significant at *p* value < 0.05

## Discussion

This study aimed to assess the level of professional nursing competency of nurses who work at a tertiary hospital. Using the NPC Scale, the study’s findings shed light on the degree of self-reported professional competence among nurses working in a tertiary hospital in the West Bank. The results could be applied to raise the standard of patient care and healthcare services by pointing out areas that need improvement in the nursing clinical field, education, and training programs. This study contributes to the existing body of knowledge on the level of professional competence among nurses on the West Bank.

A total of 206 nurses participated in the study. Most of the respondents were male. The study showed no significant differences between males and females in terms of their level of professional competence; this was also noted in a study in which gender was not significantly related to professional competence [23]. In contrast, a study conducted on nurses’ competency in the Saudi Arabian healthcare context showed that male participants demonstrated superior self-reported competency assessment compared to female participants [24].

On the other hand, this study showed that years of experience do not affect the competency level, in contrast to a Japanese study in which the nursing competence levels are affected by the clinical experience. high competency level among newly hired nurses and junior nurses [25]. Also, a systematic review in Iran indicated that clinical experience of more than nine years affects the competency level [26].

The educational level of nurses in this study revealed no discernible relationship with their competence, and this is supported by the study of S-O Kim and Y-J Choi [27] contradicting the study of Z Nabizadeh-Gharghozar, NM Alavi and NM Ajorpaz [28] that correlates the educational level with competence level This discrepancy in results underscores the necessity for further exploration to understand the nuanced relationship between education levels and nursing competencies [29]. While a notable correlation emerged in this study between the number of workshops attended by nurses and their competence levels across all competency domains, a recent study in Japan showed that attending a two-day international outreach seminar provided participants with valuable and current knowledge regarding the competency of nurse educators. They developed a heightened awareness of the shifts in their self-efficacy as educators [30]. Additionally, Egyptian studies concluded that workshops had a beneficial impact on enhancing the knowledge, collaboration skills, and overall performance of both head and staff nurses [31].

According to our study, nurses exhibited a very good level of the total professional competency level. This result was supported by a study conducted in Iran which reported that nurses had a very good competency level [32]. Delving into the assessment of competency sub scores our study excelled in evaluating the competency of providing nursing care and helping patients was very good and the same with the result of a Turkish study that assessed the caring and helping competency level of 243 nurses in a university hospital [33],. Similarly, participants showed a very good competency level in handling technology and advanced medical machines, which affirms the growing integration of technology in nursing practice [34]. The “Care Pedagogics” competency underscores the crucial role of nurses in educating and supporting patients and their families, which is consistent with the findings of other related research [32, 35] These results emphasize the ongoing need to prioritize clinical proficiency in nursing education and practice [17].

Moving into the sphere of “Documentation and Administration of Nursing Care”, nurses in our study had a very good competency level in developing a collaborative care plan and documentation skills echoing the significance of nursing documentation and administration for ensuring high-quality patient care [36], Additionally, the participant’s had very good competency in “Development, Leadership, and Organization of Nursing Care” which underscored the nursing abilities to lead and supervise teamwork and prioritize care aligning with the findings of various studies supporting this notion [37–39].

According to the study’s findings, nurses generally do well in areas including nursing care, value-based nursing care, technical and medical treatment, and administration and documentation. In contrast, a study highlighting possible areas for focused improvement in nursing practice and education found that nurses tended to report lower competence scores in the areas of development, leadership, organization of nursing care, and care pedagogy [40].

Furthermore, it’s critical to stress how important it is for nurses to maintain quality of life. To guarantee that high-quality care is provided, initiatives to enhance the quality of life for nurses must be initiated. When creating projects and programs to improve nurses’ competence, nurse managers should take the results into account [41] and use reflective learning, which can help both new and novice nurses because it will help them develop a good self-perception of their competence [42].

### Implications

#### Theoretical implication

The results of this study contributed to the theoretical understanding of factors affecting the professional competencies of nurses. The notable correlation between the number of continuing education activities attended by nurses and the level of nurses’ performance implies that professional development programs have a pivotal role in enhancing and improving nurses’ competency in several domains. This result goes in alignment with the “ Novice to Expert” theory of Benner and empirically supports the crucial role of ongoing education in improving and supporting nurses to advance their professional competencies and growth. On the other hand, the multidimensional aspects of the applied professional competency in this study such as the documentation, value-based, and technical aspects all act as factors that shape the wholistic approach to nursing care and the nature of the nurse’s practices which require more wholistic evaluation method for nurses competencies.

#### Managerial implications

The majority of nurses in this study were professionally competent which indicates that available ongoing educational activities were valuable and effective opportunities to promote nurses’ competencies. However, having 15 nurses with a 60% competency level highlights the importance of structuring more need-sensitive ongoing education programs and interventions. On the other side, the strong relationship between attending continuing education activities and a higher level of nurses competencies across various domains underscores the pivotal role of offering equal and efficient opportunities for attending and joining the available activities, also health care institutions may need to invest more effectively in promoting and supporting goal-based, and need-sensitive professional development programs in their setting to get more competent and qualified nurses and subsequently high-quality patient care.

Additionally, the study findings highlight that subdomains and sub-dimensions of nurses’ practices such as care and value-based aspects and documentation and administration have essential roles in formulating the overall professional competency level of nurses. This guides the nurse managers and leaders to establish a more uniform performance appraisal process to evaluate nurses’ practices. This would enable nurse leaders to effectively identify any practice gaps and areas for improvement and this helps them to efficiently utilize resources to provide the required learning activities and offer equal chances for improving nurses’ performance. Determining a nurse’s areas of competence can serve as a reference to guide the hiring process of new staff.

The outcome of this study can steer the adoption of robust ongoing education such as mentorship and preceptorship programs, cross-training programs, in-service clinical training, and other professional development opportunities to facilitate the rapid transition of newly hired nurses from beginner level to more competent and proficient nurses.

A trustworthy tool that recognizes a nurse’s level of professional competency can assist policymakers, managers, and nurse educators in defining the skills, knowledge, and attitude necessary for nurses to perform their jobs. The findings of this research can also be used to create more customized and goal-oriented professional development programs, pinpointing areas of best practices that require improvement and investigating the necessary methods and resources to improve nurses’ competencies while emphasizing evidence-based practices. Additional research examining the relationships between nursing competence and patient outcomes may be beneficial in enhancing best practices for nurses.

### Limitations

1. The sample was convenient with 206 nurses participating in the study, which potentially limits the generalizability of the findings to a broader nursing population. Additionally, the overrepresentation of male respondents might skew the results and not accurately reflect the gender balance in nursing.
2. The data primarily relied on self-reported measures, which might introduce response bias and subjectivity. Objective assessments or external evaluations of competence could enhance the validity of the findings.
3. The study utilized a cross-sectional design, providing a snapshot of competence at a specific time. Longitudinal studies tracking nurses’ competence over time could offer a more comprehensive understanding of competence development.
4. The study did not comprehensively explore other potential influencing factors, such as workload, staffing ratios, or specific training programs attended by participants. These factors can significantly impact nursing competence and have not been thoroughly investigated.

## Conclusions

In this study, we meticulously evaluated the professional competence levels of 206 nurses employed in a tertiary hospital setting. The findings revealed that the professional competence level was moderately high among the participants. However, it is noteworthy that while a significant portion of nurses demonstrated high levels of competency, a considerable number still exhibited competence levels below the desired threshold, with 15 nurses scoring below 60%.

Our comprehensive assessment encompassed various competency areas, shedding light on specific domains where nurses warrant focused attention. Notably, the domain of “Development, Leadership, and Organization of Nursing Care” exhibited a slightly lower average competency level (78%). Therefore, it is important to promote nurse’s knowledge and skills in the domain of leadership and management principles.

Attending workshops plays a significant role in improving nurses’ competencies, especially the competency in documentation, management, and leadership skills. So, investments in providing a well-designed workshop with a clear outcome are essential to affect the level of competence among nurses. Moreover, the findings underscored the importance of continuing education and training programs to foster nurses’ competency, and subsequently, improve the quality of patient care.

In conclusion, this study provides valuable insights into the nuanced landscape of nursing competence, highlighting both areas of strength and opportunities for improvement. Moving forward, healthcare institutions and educational bodies must prioritize ongoing education and targeted interventions aimed at fortifying nursing competencies for the betterment of patient care.

### Recommendations

1. Healthcare institutions should invest in continuous training programs, and make sure they are goal-directed and have outcomes related to improving the staff knowledge as well as the skills and contributing to improving the competency level.
2. More emphasis needs to be placed on the development of leadership and management abilities. The ongoing educational initiatives should arrange more organized and impactful workshops and training programs to enhance this particular aspect, which in turn will have a direct impact on the competencies of nurses.
3. Encourage collaboration between academia and healthcare institutions to conduct research focused on nursing competence to disseminate competency development to regional policymakers and initiate training programs and the potential implementation of a “clinical ladder” system for nurses.
4. Future studies should involve larger and more diverse samples across various healthcare settings to capture a more representative picture of nursing competence. Ensuring a balanced gender representation among participants would yield more comprehensive insights.
5. Complementing self-reported measures with objective assessments or observations of nursing practices could enhance the robustness and validity of the findings. Qualitative interviews or focus groups might provide richer insights into the factors affecting nursing competence.
6. Conducting longitudinal studies to track nurses’ competence development over time would offer a deeper understanding of competency growth and fluctuations throughout a nurse’s career trajectory.
7. Future research should further explore the various factors influencing nursing competence, including workload, staffing, continuing education programs, and the impact of specific training initiatives on competence levels.
8. Implementing targeted interventions or training programs and evaluating their impact on nursing competence could provide valuable insights into effective strategies for enhancing nursing competency.
9. Collaborating with multiple healthcare facilities or employing a multicenter approach would provide a more extensive dataset and facilitate comparisons between institutions, enriching the understanding of nursing competence on a broader scale.

## Data Availability

Primary research article or the corresponding author are the sources of all data. Data that supports the results of the manuscript is provided within the manuscript.

## Abbreviations

AAUP: Arab American University
IRB: Institutional Review Board
NNUH: An-Najah-National University Hospital
NPCS: Nurse Professional Competence Scale
SPSS: Statistical Package for the Social Sciences
WHO: World Health Organization
